# Atrial stunning as a risk factor for thromboembolic events after cardioversion of atrial flutter

**DOI:** 10.1093/ehjcr/ytag127

**Published:** 2026-02-26

**Authors:** Leonie Hahn, Alvaro Petersen-Uribe, Thomas Nordt, Elke Roser

**Affiliations:** Department of Cardiology and Angiology, Stuttgart Hospital, Klinikum Stuttgart, Stuttgart 70174, Germany; Department of Cardiology and Angiology, Stuttgart Hospital, Klinikum Stuttgart, Stuttgart 70174, Germany; Department of Cardiology and Angiology, Stuttgart Hospital, Klinikum Stuttgart, Stuttgart 70174, Germany; Department of Cardiology and Angiology, Stuttgart Hospital, Klinikum Stuttgart, Stuttgart 70174, Germany

## Case description

An 83-year old female patient referred recent onset dyspnoea, reduced exercise capacity and fatigue. The ECG showed a recurring atrial flutter with a heartrate of 122 bpm, as it had been treated with a cavotricuspid-isthmus ablation 3 months earlier. Long-term anticoagulation with direct oral anticoagulants (DOACs), namely 5 mg Apixaban every 12 h, had been taken consistently. Due a fall months earlier, which led to a chronic subdural haematoma, a dose reduction had been temporarily performed. Subsequently, an approach involving rhythm control through electrical cardioversion and a left atrial appendage (LAA) occlude was suggested and agreed upon by the patient.

**Figure 1:**
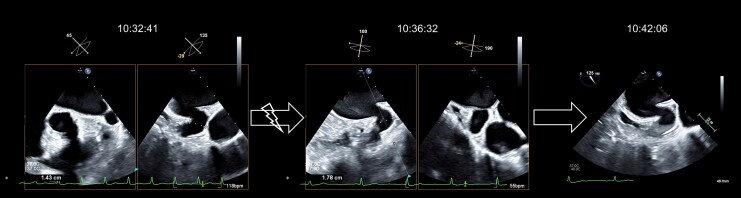
Transoesophageal view of a pre-thrombotic formation in the LAA after electrical cardioversion.

The day of the procedure, the oral anticoagulation was paused solely the morning of the LAA occlusion. A transoesophageal echocardiography showed no intracardial thrombi, particularly in the LAA. Restoration of the sinus rhythm was achieved with 70J of biphasic current. No intracardial catheters were in place at the moment of the cardioversion. Within three to four minutes, spontaneous echo contrast could be observed in the left atrium, a phenomenon associated with atrial stunning.^1^ Shortly after, the LAA showed a pre-thrombotic formation, as shown in the *Figure 1*. A stunned atrium is known to undergo a paradoxical decrease in atrial and LAA function, leading to a reduced LAA emptying velocity, in turn increasing the risk of new thrombus formation.^2^ Unfractionated heparin was immediately administered intravenously, followed by the morning dose of her DOAC. The planned LAA occlusion was cancelled. After an uneventful period of inpatient observation, the patient was discharged. In spite of no PW-Doppler measurements, a visual decrease of the LAA contractility was observed after cardioversion. This case underscores the critical importance of periprocedural anticoagulation in patients undergoing cardioversion for atrial fibrillation or atrial flutter. Even brief interruptions in anticoagulation during the vulnerable post-cardioversion period may increase the risk of thromboembolic events.^3^

**Consent:** Written informed consent for submission and publication of this case report including images has been obtained from the patient in line with COPE guidance.

## Data Availability

The data underlying this article will be shared on reasonable request to the corresponding author.
